# Parallel Patterns of Morphological and Behavioral Variation among Host-Associated Populations of Two Gall Wasp Species

**DOI:** 10.1371/journal.pone.0054690

**Published:** 2013-01-21

**Authors:** Scott P. Egan, Glen R. Hood, Gabriel DeVela, James R. Ott

**Affiliations:** 1 Department of Biological Sciences, University of Notre Dame, Notre Dame, Indiana, United States of America; 2 Advanced Diagnostics and Therapeutics Initiative, University of Notre Dame, Notre Dame, Indiana, United States of America; 3 Population and Conservation Biology Program, Department of Biology, Texas State University – San Marcos, San Marcos, Texas, United States of America; University of Arkansas, United States of America

## Abstract

A powerful approach to address the general factors contributing to ecological speciation is to compare distantly related taxa that inhabit the same selective environments. In this design, similarities among taxa can elucidate general mechanisms of the process whereas differences may uncover specific factors important to the process for individual taxa. Herein, we present evidence of parallel patterns of morphological and behavioral variation among host-associated populations of two species of cynipid gall wasps, *Belonocnema treatae* and *Disholcaspis quercusvirens*, that each exhibit a life cycle intimately tied to the same two host plant environments, *Quercus geminata* and *Q. virginiana*. Across both gall-former species we find consistent differences in body size and gall morphology associated with host plant use, as well as strong differences in host plant preference, a measure of habitat isolation among populations. These consistent differences among taxa highlight the important role of host plant use in promoting reproductive isolation and morphological variation among herbivorous insect populations–a prerequisite for ecological speciation.

## Introduction

Throughout the modern synthesis, biologists described a central role for ecological adaptation in the speciation process [1]–[3]. However, it was not until the recent renaissance of empirical studies that specific ecological barriers have been shown experimentally to contribute to reproductive isolation through a process termed ecological speciation [4]. Specifically, ecological speciation describes a process in which reproductive isolation evolves as a by-product of divergent natural selection among environments [5]. Examples of ecological speciation are present across a diverse set of taxa (e.g., insects [6]–[10], snails [11], fishes [12], [13], and plants [14]). These studies have documented that a diversity of prezygotic and postzygotic reproductive barriers can arise as a result of divergent ecological adaptation [4], [15]. Barriers examined include temporal isolation [16], sexual isolation [17], cryptic isolation [18], and extrinsic (ecological) postzygotic isolation [19], [20].

To determine the generality of environment-specific factors that may promote (or impede) ecological speciation, it is necessary to examine ecological divergence across multiple taxa experiencing similar environments [21]. Thus far, the majority of studies addressing parallel ecological speciation have compared geographically separate populations of individual species in different versus similar environments [7], [9], [13], [22], [23]. These studies have suggested a central role for divergent selection in promoting speciation [4]. A complementary and powerful approach to understand the process of ecological speciation is to examine more distantly related taxa that inhabit the same selective environments. Similarities among taxa in their response to divergent selection may then suggest specific hypotheses that can then be tested to elucidate general mechanisms of the process, whereas differences may uncover specific factors important to the biology of each species. Evidence for parallel ecological speciation produced by comparing more distantly related taxa is rare but includes studies of multiple lizard species inhabiting two soil color habitats [24] and multiple herbivorous insect species inhabiting similar sets of host plants [25]–[28].

**Figure 1:**
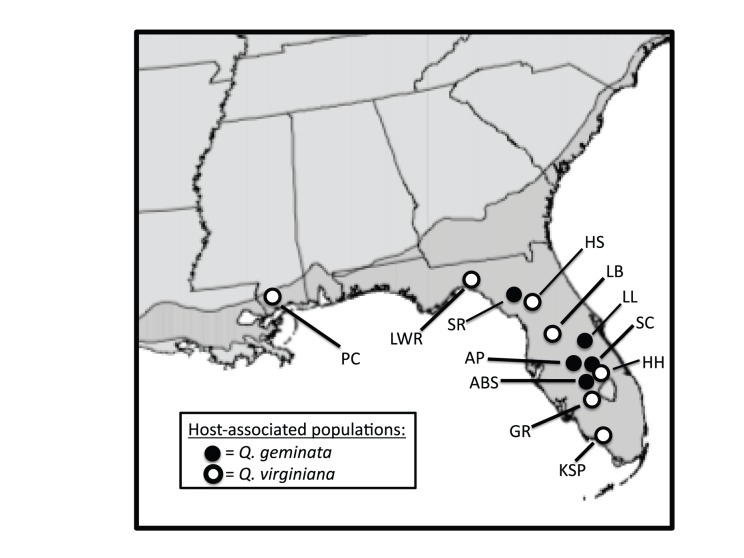
Sampling localities across southeastern USA. Map of host-associated populations of two species of gall formers on live oak used in the present study. Darker grey shading along coastal USA and Florida illustrates the general distribution of both host plants. See Tables 2 and 3 for population abbreviations.

Across the southeastern United States a diverse community of host-specific gall formers (Hymenoptera: Cynipidae) inhabits two sister species of live oak, *Quercus virginiana* and *Q. geminata.* These oak species overlap in geographic range (Figure 1) but occupy different microhabitats. Specifically, *Q. virginiana* occurs in moister, more nutrient rich, and higher pH sites than *Q. geminata* [29]. The two species also differ in leaf morphology, flowering time, and growth and photosynthetic rates [29]. Thus within each species of cynipid, populations of gallers resident on the alternative oak species may experience different environmental challenges and thus may undergo divergent natural selection related to the host plant and (or) the habitat occupied by the alternative host plants. With a minimum of eight gall wasp species [30] spanning six different genera [31] inhabiting both oak species, this system provides an opportunity to test for the parallel effects of divergent host use across a community of gall formers.

Herein we examine evidence for parallel host-associated differences in adult morphology and behavior among multiple populations for each of two cynipid species, *Belonocnema treatae* [32] and *Disholcaspis quercusvirens* [33], that each inhabit *Q. virginiana* and *Q. geminata*. For each cynipid species we examine a main prediction of ecological speciation theory: allopatric populations inhabiting different host plant species will exhibit greater differences in ecologically important traits (e.g., body size [34] and gall morphology [35]) and express greater reproductive isolation (here, habitat isolation due to host preference) than will allopatric populations inhabiting the same host plant. Thus, we specifically sampled both gall-former species from distinct allopatric stands of each host plant species (Figure 1). By comparing multiple allopatric pairs of populations within each gall wasp species that occur in ecologically similar habitats (i.e., host plant species), we can control for host plant independent divergence (i.e., due to genetic drift, sexual conflict, and certain forms of sexual selection) whereas pairs of populations in ecologically different habitats may diverge due to any of these processes as well as from host plant associated divergent selection [7], [22].

## Methods

### (a) Ethics Statement

A permit to perform fieldwork and make collections at Archibold Biological Station (ABS), Florida, was approved prior to our work. No other permits were required as all other field sites were along public roadways in Florida and Mississippi, USA. Moreover, collections did not involve any endangered or protected species.

### (b) Study System and Sampling

*Belonocnema treatae* (Mayr) and *Disholcaspsis quercusvirens* (Ashmead) (Hymenoptera: Cynipidae) are both ecologically specialized gall formers that attack certain species of ‘live oaks’ in the genus *Quercus* [9], [10], [30], [31], [36], [37]. The ‘live oaks’ (series *Virentes*) are a lineage of five to seven oak species forming a nearly continuous distribution from the tropics (Costa Rica) to the temperate regions (southeastern United States) [29]. The two sister species, *Q. virginiana* and *Q geminata*, overlap geographically in the southeastern United States, where *Q. geminata* is restricted to this region, but *Q. virginiana* stretches further up the Atlantic coast to Virginia and further west and south along the Gulf coast into Texas and Mexico [29]. We have previously demonstrated host specificity among populations of *B. treatae* on *Q. virginiana* and *Q. geminata*, as measured by host preference [9], [10].

**Figure 2:**
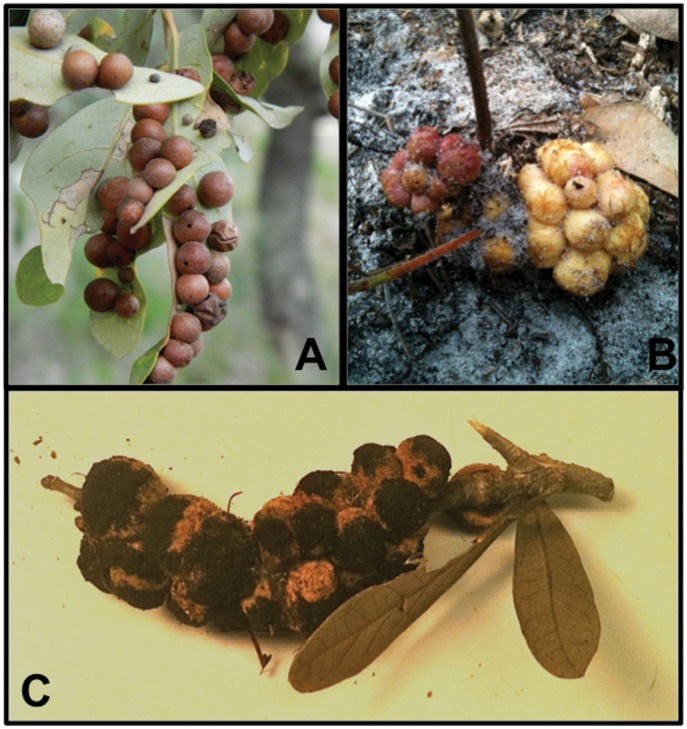
Galls induced on live oaks by *B. treatae* and *D. quercusvirens*. (A) Spherical, unilocular leaf galls housing the asexual generation of *B. treatae* on the host plant *Q. virginiana*; (B) multi-chambered root gall housing the sexual generation of *B. treatae* on the host plant *Q. geminata*; and (C) compound stem gall housing the asexual generation of *D. quercusvirens* on the host plant *Q. virginiana*.

Each gall wasp species exhibits cyclic parthenogenesis (heterogony) whereby spatially and temporally segregated asexual and sexual generations alternate to complete a yearly life cycle [37], [38]. Specifically, the asexual generation of *B. treatae* develops within single-chambered spherical leaf galls (Figure 2A) on the underside of newly flushed leaves during the summer and fall and emerges in the winter. The sexual generation then develops within multi-chambered galls on oak roots (Figure 2B). Males and females emerge in the spring corresponding with spring bud break [37] and (or) leaf flushes following defoliation by insect herbivores [39]. Similarly, the asexual generation of *D. quercusvirens* develops within woody compound stem galls (Figure 2C) during early shoot expansion in the summer and fall and emerges in the winter [30], [38]. The woody compound stem galls of the asexual generation comprise clusters of many single-chambered globular galls. While detailed information is lacking on the sexual generation of *D. quercusvirens*, Platt [38] reports that the sexual generation develops within bud galls initiated beneath bud scales and males and females emerge in the spring. Comparisons presented in this study involve both the asexual and sexual generations of *B. treatae* and the asexual generation of *D. quercusvirens*.

We collected *B. treatae* root galls in the spring of 2010 and 2012 and *B. treatae* leaf galls and *D. quercusvirens* stem galls in the fall of 2010. Adult gall formers were reared from galls housed individually by cynipid species, host plant, and locality and husbanded under common conditions (12∶12 light:dark, 23°C) at the University of Notre Dame. Host preference assays were performed within 48 hours of emergence in the same conditions as the rearing environments.

More generally, given the diverse community of cynipid gall formers reported in the historical literature on one or both of these two sister oak species, the patterns revealed for the two species studied here can generate a framework for further testing across all species (26, 40).

Thus, the full suite of cynipid species on each host plant at each locality was collected and is documented in Table 1.

**Table 1:** Cynipid gall wasp species that inhabit the live oaks, *Q. virginiana* and *Q. geminata*, across the southeastern USA.

Cynipid species	*Q. virginiana*	*Q. geminata*	Reference
*Andricus quercusfoliatus* (Ashmead 1881)	HS, LWR, HH, LB	LL, ABS, AP, SR	[33]
*Andricus quercuslanigera* (Ashmead 1881)	HS, LWR, HH, PC,	AP, ABS, SR	[33]
*Bassettia pallida* (Ashmead 1896)	HH	ABS	[67]
*Belonocnema treatae* (Mayr 1881)	HS, LB, PC, HH, KSP, GR	AP, ABS, SR, SC, LL	[32], [37]
*Callirhytis quercusbatatoides* (Ashmead 1881)	HH, HS	ABS, SR	[33]
*Disholcaspis quercusvirens* (Ashmead 1881)	HH, HS, LB, LWR,	LL, ABS, SR, SC	[33]
*Neuroterus christi* (Melika & Abrahamson 1997)	HH	ABS, SC	[31]
*Neuroterus quercusminutissimus* (Ashmead 1885)	HS, LB	LL, AP, ABS, SR	[68]

Cited references list host use as one or both oak species; however, we verified that each gall former species inhabits each host plant species in multiple localities (see Tables 2 and 3 for locality abbreviations and Figure 1 for geographic distribution).

### (c) Cynipid Body Size and Gall Morphology

For both species, the body size of adult wasps developing on each host plant species was indexed by measuring the length of the right hind tibia. For *B. treatae*, we compared the body size of asexual generation adults among populations distributed within and between host plant species. This analysis complements previous comparisons of the sexual generation [9]. For *D. quercusvirens*, we compared the body size of asexual generation adults among populations distributed within and between host plant species. We then compared measures of gall morphology among host-affiliated populations of *B. treatae* for both the sexual and asexual generations. Following emergence of the sexual generation, we dissected and counted the number of chambers within each multi-chambered root gall (Figure 2B). To compare gall size among populations of the sexual generation of *B. treatae* we combined new data from collections made in 2010 and 2012 with previous data collected in 2010 and thus expanded the number of populations and sample sizes reported previously by Egan et al. [9]. Following emergence of the asexual generation, the diameter of each spherical leaf gall (Figure 2A) was measured with digital calipers. Gall morphology for *D. quercusvirens* was compared among populations for the asexual generation. For each compound stem gall both the number and the average diameter of each individual stem gall forming the compound gall was measured (Figure 2C).

### (d) Host Preference Assays

For both gall-former species, host preference trials were conducted within 25×8 cm clear-plastic cups stocked with a fresh cutting of each host plant (*Q. virginiana* and *Q. geminata*) collected within the native range of the cynipids. A single individual female of either *B. treatae* or *D. quercusvirens* was aspirated into each cup and then observed at 5-minute intervals for 1 hour for a total of 12 observations. At each time point, we recorded the location (on *Q. virginiana*, on *Q. geminata*, or on the cup) of each individual. Host preference was calculated for each individual as the relative time spent on one host plant species divided by the total time spent on both host plants during the trials (e.g., for *Q. virginiana* = [# of observations on *Q. virginiana*]/[# of observations on *Q. virginiana*+# of observations on *Q. geminata*]). We performed, in total, 116 preference assays on sexual generation *B. treatae* females distributed across six populations (Table 2) and 107 preference assays for asexual *D. quercusvirens* distributed across five populations (Table 3). The host plant preference of sexual generation female *B. treatae* has been previously documented by Egan et al. [10].

**Table 2:** Populations of *B. treatae* associated with *Q. geminata* (*Qg*) and *Q. virginiana* (*Qv*) used in the present study, along with study site location and the population means ± SE and sample sizes (in parentheses) for each trait measured (HTL = hind tibia length [asexual generation], CPG = chambers per root gall, LGD = leaf gall diameter, and HP = host preference of sexual generation females for *Q. virginiana* [ = 1 − host preference for *Q. geminata*]).

Population	Host plant	Latitude	Longitude	HTL (mm)	CPG	LGD	HP
Avon Park (AP)	*Qg*	27° 36′ 00′′ N	81° 30′ 42′′ W	1.22±0.01(15)	8.53±1.55(16)	6.45±0.09(352)	0.19±0.03(16)
Lake Lizzie (LL)	*Qg*	28° 13′ 39.6′′ N	81° 10′ 47.9′′ W	1.20±0.02(12)	n/a	5.88±0.04(999)	n/a
Archibold Biol. Station (ABS)	*Qg*	27° 10′ 57′′ N	81° 21′ 08′′ W	1.38±0.03(20)	9.92±1.31(37)	6.22±0.04(820)	0.21±0.03(22)
Scrub field (SC)	*Qg*	27° 30′ 48′′ N	81° 20′ 16′′ W	n/a	7.19±1.88(10)	n/a	0.21±0.03(20)
Hickory Hammock (HH)	*Qv*	27° 24′ 09′′ N	81° 06′ 42′′ W	1.19±0.02(20)	1.47±0.68(73)	5.50±0.04(760)	0.85±0.03(22)
High Springs (HS)	*Qv*	29° 50′ 37.9′′ N	82° 37′ 54.6′′ W	1.05±0.02(12)	n/a	4.93±0.04(289)	n/a
Leesburg (LB)	*Qv*	28° 40′ 2.5′′ N	81° 51′ 11.7″ W	1.11±0.02(9)	n/a	4.92±0.06(204)	n/a
Picayune (PC)	*Qv*	30° 31′ 37.8′′ N	89° 40′ 52.4′′ W	1.08±0.04(15)	3.26±2.12(6)	5.06±0.05(326)	n/a
Koreshan State Park (KSP)	*Qv*	26° 26′ 04′′ N	81° 48′ 56′′ W	n/a	1.83±0.76(17)	n/a	0.72±0.03(18)
Gatorama (GR)	*Qv*	26° 55′ 30′′ N	81° 18′ 44′′ W	n/a	1.01±0.49(19)	n/a	0.67±0.03(18)

**Table 3:** Populations of *D. quercusvirens* associated with *Q. geminata* (*Qg*) and *Q. virginiana* (*Qv*) used in the present study, along with study site location and the population means ± SE and sample sizes (in parentheses) for each trait measured (HTL = hind tibia length [asexual generation], GPC = galls per compound stem gall, MGD = mean gall diameter per individual gall that forms compound gall, HP = host preference of the asexual generation females for *Q. virginiana* [ = 1 − host preference for *Q. geminata*]).

Population	Host plant	Latitude	Longitude	HTL(mm)	GPC	MGD	HP
Lake Lizzie (LL)	*Qg*	28° 13′ 39.6′′ N	81° 10′ 47.9′′ W	1.48±0.01 (38)	9.50±2.35(12)	10.32±0.19(192)	0.32±0.03(17)
Archibold Biol. Station (ABS)	*Qg*	27° 10′ 57′′ N	81° 21′ 08′′ W	1.53±0.01(28)	6.48±1.79(21)	8.03±0.18(124)	0.41±0.04(27)
Sewanee River at Branford (SR)	*Qg*	29° 57′ 18.4′′ N	82° 55′ 43.7 ′′W	1.56±0.03(34)	7.39±0.81(28)	8.41±0.16(207)	n/a
Scrub field (SC)	*Qg*	27° 30′ 48′′ N	81° 20′ 16′′ W	1.61±0.02(12)	8.31±2.27(13)	9.91±0.24(79)	0.37±0.04(25)
Hickory Hammock (HH)	*Qv*	27° 24′ 09′′ N	81° 06′ 42′′ W	1.41±0.02(9)	6.06±0.96(16)	6.49±0.16(104)	0.65±0.05(19)
High Springs (HH)	*Qv*	29° 50′ 37.9′′ N	82° 37′ 54.6′′ W	1.37±0.02(8)	7.96±1.45(27)	5.55±0.08(234)	n/a
Leesburg (LB)	*Qv*	28° 40′ 2.5′′ N	81° 51′ 11.7″ W	1.33±0.03(12)	n/a	n/a	n/a
Luther Wilson Road (LWR)	*Qv*	30° 19′ 4.8′′ W	83° 45′ 38.3′′ W	1.39±0.01(30)	9.61±1.55(26)	6.91±0.10(252)	0.67±0.05(19)

### (e) Statistical Analyses

For each gall-former species, populations on the same host plant species offer comparisons between ecologically similar environments (hereafter, “same-host pairs”) while pairs of populations on different host plants offer comparisons between ecologically dissimilar environments (hereafter, “different-host pairs”). To test for differences in body size and gall morphology among host-associated populations within each species, we generated datasets in the form of matrices of pairwise comparisons among same-host and different-host populations. To compare elements between matrices, Mantel tests [41] were run in the “ecodist” package in R version 2.11.1 with 10,000 randomizations and one-tailed hypothesis testing.

To test for differences in host plant preferences of individual females among populations, we conducted an ANOVA on individual relative preference for *Q. virginiana* ( = 1 − preference for *Q. geminata*), with population treated as a random effect, followed by a Tukey’s HSD test to compare means among populations. Since ANOVA determines only whether different populations vary from each other, we then compared each population’s relative preference for its native host to a value of 0.5 by means of a one-sample *t*-test. The value 0.5 indicates no preference, that is, equal time spent on each of the two host plants, whereas a significant difference implies a preference for a specific host plant. All ANOVA and *t*-tests were run in the program JMP version 5.0.1a (SAS Institute).

## Results

### (a) Parallel Patterns of Phenotypic Divergence

**Figure 3:**
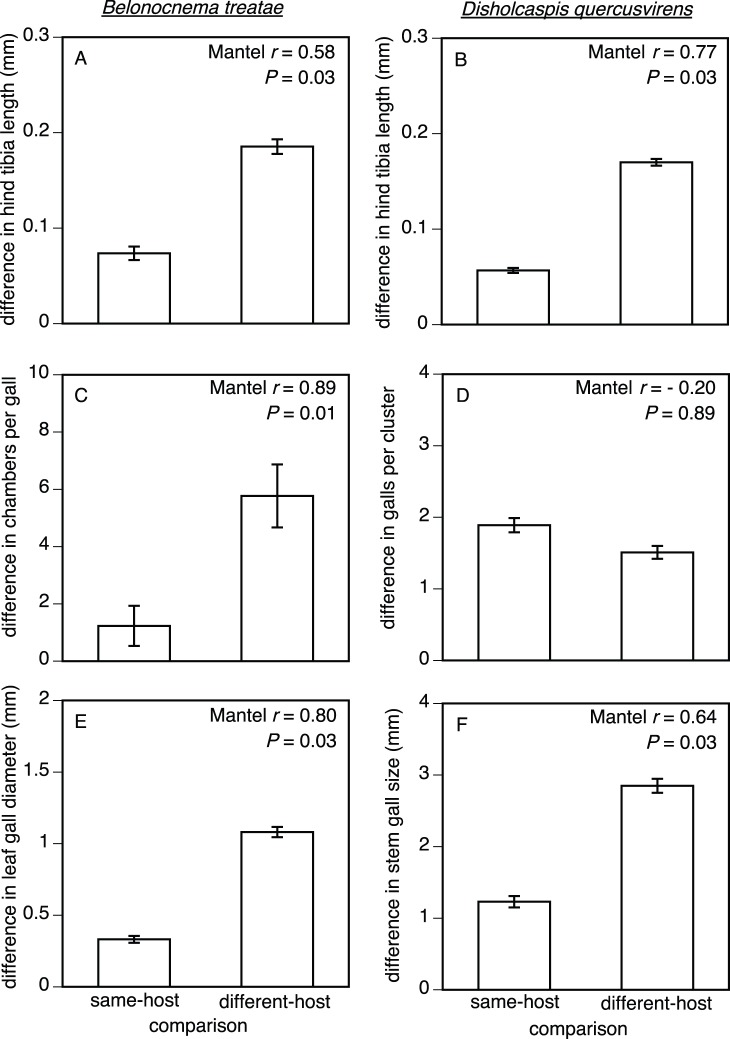
Parallel phenotypic patterns among populations of *B. treatae* (left) and among populations of *D. quercusvirens* (right) across the same two host plants, *Q. virginiana* and *Q. geminata*. All values compare mean (± SE) of pairwise differences among populations for each species: (A & B) illustrate body size differences, (C & D) illustrate gall structure differences, and (E & F) illustrate gall size differences. See Tables 2 and 3 for sample sizes per population.

Adult *B. treatae* from the asexual generation exhibited greater differences in body size among populations in different-host comparisons than in same-host comparisons (Mantel *r* = 0.58, *P* = 0.031; Figure 3A). Differences among populations in chamber number per root gall of the sexual generation (Mantel *r* = 0.89, *P* = 0.012; Figure 3C) and diameter of the leaf galls produced by the asexual generation (Mantel *r* = 0.80, *P* = 0.028; Figure 3E) were also associated with different host plant use. Thus, asexual generation populations of *B. treatae* on *Q. geminata* produced larger females and larger leaf galls whereas sexual generation populations of *B. treatae* produced root galls with more chambers on *Q. geminata* than did sexual populations of *B. treatae* on *Q. virginiana* (Table 2).

Populations of *D. quercusvirens* exhibited greater differences in asexual female body size in the different-host comparisons than in same-host comparisons (Mantel *r* = 0.77, *P* = 0.028; Figure 3B) but did not exhibit differences in galls per compound gall associated with host use (Mantel *r* = −0.20, *P* = 0.885; Figure 3D). The average size per individual gall within a compound gall was associated with host use (Mantel *r* = 0.64, *P* = 0.031; Figure 3F). Thus, populations of *D. quercusvirens* on *Q. geminata* produced larger asexual females and larger individual galls within clusters than did populations of *D. quercusvirens* on *Q. virginiana* (Table 3).

### (b) Parallel Patterns in Habitat Isolation

**Figure 4:**
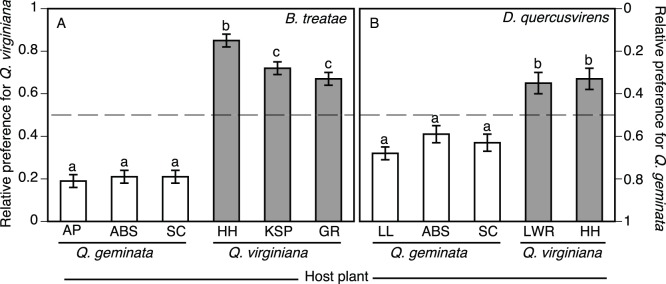
Parallel behavioral patterns in host preference among (A) *B. treatae* and (B) *D. quercusvirens* populations associated with *Q. virginiana* or *Q. geminata*. Mean (± SE) illustrates the proportion of time spent on *Q. virginiana* by each population; however, note reversed *y*-axis on right (mean time on *Q. geminata* = 1– mean time on *Q. virginiana*). The dashed line highlights no preference (defined as 50% of time spent on each host). Each population differs significantly from 50% (*P*<0.001). See Tables 2 and 3 for locality information and sample sizes per population. Letters above bars denote significant differences among means from Tukey’s HSD test (*P*<0.05).

Sexual generation female *B. treatae* exhibit geographic variation in relative host plant preference among populations (ANOVA on female host preference: *F*_5,110_ = 113.4, *P*<0.001) that is associated with host plant of origin (Tukey’s HSD test: [AP = ABS = S]<{[GR = KSP]<HH}, *P*<0.05; Table 2, Figure 4A). All populations differed from a no preference expectation of equal time spent on each host plant (*t*-test of population mean versus 0.50: all *t*-values >5.40, df ranged from 29–41, all *P*-values <0.001). Further details can be found in Egan et al. [10].

In parallel, *D. quercusvirens* exhibited geographic variation in relative host plant preference among populations as evidenced by a significant population effect (ANOVA: *F*_4,99_ = 13.89, *P*<0.001). This difference was associated with the population’s host plant of origin (Tukey’s HSD test: [LL = S = ABS]<[HH = LWR], *P*<0.05; Table 3, Figure 4B). Each population expressed preference for its native host, as all populations differed from a no preference expectation of equal time spent on each host plant (*t*-test of population mean versus 0.50: LWR *t_df_*_ = 18_ = 3.40, *P* = 0.002; ABS *t_df_*_ = 26_ =  −2.44, *P* = 0.011; HH *t_df_*_ = 16_ = 2.78, *P* = 0.007; LL *t_df_*_ = 16_ =  −5.19, *P*<0.001; SC *t_df_*_ = 22_ =  −3.76, *P* = 0.001).

## Discussion

A consistent and repeated pattern of variation in trait values among populations inhabiting the same environmental gradient suggests an important role for ecology in generating morphological variation and promoting reproductive isolation among populations–a prerequisite for ecological speciation [7], [8], [21], [24], [26]. However, evidence for similar patterns of variation among populations within two or more species distributed across the same environmental gradient is uncommon, in part because it is rarely tested [24]–[28]. In the present study we provide evidence from two cynipid gall wasp species representing different genera that differences in host plant use within each species are associated with parallel differences in adult and gall morphology between cynipid species. Previously, we demonstrated that partial reproductive isolation, arising as a by-product of significant habitat preference, is exhibited by populations of *B. treatae* [9], [10]. Our results support the perspective that patterns of host plant use play a central role in promoting both morphological variation and reproductive isolation among populations of herbivorous insect species.

The morphological variation exhibited by cynipid populations on each host plant may be explained by the hypothesis of heritable adaptive divergence among populations. Alternatively, non-genetic effects of the host environment represents a competing hypothesis to explain the observed patterns as all individuals measured in the present study developed on their native hosts. Reciprocal transplant experiments, repeated across populations of each gall-former species, will be needed to distinguish genetic and non-genetic contributions to the observed differences in trait expression and to assess the adaptive nature of trait variation through measurements of the mean fitness of each population on the two host plant species. Our results justify further exploration of the causal basis of variation in morphology (and behavior) within populations of *B. treatae* and *D. quercusvirens* and suggest that the hypothesis of host plant driven divergence should be examined for the full suite of cynipid species that comprises the community of gall formers on these two species of live oak. While the present study was not designed to evaluate the relative contributions of adaptive differentiation and plasticity to observed trait variation, we note that populations of both cynipid species exhibited consistent differences across host plant populations even though there is evidence of significant variation in leaf morphology among populations of each oak species and genetic structure is present among populations of each host plant [29]. These consistent differences across variable plant populations exhibited by populations of both cynipid species argues against plasticity as the sole explanation for the patterns of morphological divergence observed within each gall former species [40].

As with patterns of variation observed for morphological traits, our results documenting parallel differences in host plant preference among *B. treatae* and *D. quercusvirens* populations may reflect genetic and (or) non-genetic contributions to variation in behavior. However, no evidence to date suggests that host plant preference in cynipids is influenced by the effects of single generation non-genetic rearing conditions [42]. More generally, the role of insect larval conditioning in influencing adult host selection behavior has been thoroughly investigated and gauged as subtle in effect and (or) rare among insects [42], including among other hymenopterans [43], [44] and herbivores [45]. However, even if the behavioral differences we observed among *B. treatae* and *D. quercusvirens* populations represent plasticity arising from rearing conditions, habitat isolation would still be predicted to contribute to reproductive isolation as individual females of both species were averse to settling on the alternative host plant and chose to spend more than twice as much time on their own host plant. Thus, regardless of the underlying basis of variation for host preference, our results suggest that choice of host plant promotes reproductive isolation among populations of each species. Lastly, our results are consistent with a growing appreciation for the role of initial environmentally induced differences (i.e. plasticity) promoting divergence and speciation [46], [47], including divergent host use among herbivorous insects [48].

### Parallel Phenotypic Differences Associated with Host Use

Phenotypic differences among populations within each species provide evidence of a response to the different host plant environments. However, the two herbivore species need not show the same pattern (i.e., one species might be bigger while the other species smaller on a specific plant species). The phenotypic differences we observed among populations within each cynipid species are in the same direction, suggesting a similar response to selection or a similar plasticity response in each gall-former species to the alternative host plant environment. To assess the general importance of each type of response in structuring phenotypic variation within and among the cynipid gall former community centered on these two oak species will require further testing of the species indicated in Table 1.

Interestingly, each of the two species tested here attack a different set of plant tissues within the host plant, but both species exhibit parallel body size differences: populations of each species are bigger on *Q. geminata* [9]. Second, and likely correlated with body size, gall size differs among populations for both species: individuals on *Q. geminata* generate larger galls than individuals on *Q. virginiana*. Differences in these traits suggest the hypothesis of adaptation to the host plant environment. The two host plants differ in a suite of morphological traits, some of which may be important to the biology of each cynipid. For example, the morphology of the leaf, which is the critical location for oviposition and gall induction for *B. treatae*, differs among the two host plants; *Q. geminata* has leaves with thicker midveins and denser trichomes and is overall more “thick and leathery” as measured by mass per area [29]. Similarly, for the stem-galling *D. quercusvirens*, host plants differ in stem diameter, root to shoot ratio, and above ground biomass [29]. Equally important to both cynipid species, host plants have been shown to differ in flowering phenology, as measured by male flowering date, and another trait associated with phenology, the number of leaf flushes when there is periodic initiation of new growth [29].

### Parallel Patterns of Habitat Isolation among Populations

Most importantly, both cynipid species exhibited habitat isolation via host plant preference. Habitat isolation arising from habitat preferences can reduce dispersal between populations inhabiting contrasting habitats and promote adaptive divergence [6], [7], [49]–[52]. Habitat preference is a critical aspect of reproductive isolation among herbivorous insect specialists, who tend to oviposit, feed, rest, develop, and mate on their host plants [53]. Habitat isolation for host-specific phytophagous insect species explicitly describes the process by which the differing habitat preferences of insect populations associated with alternative host plants reduces the frequency of encounters and thus the likelihood of mating between individuals from the differing host-associated populations. In the present study, we demonstrated that multiple populations of both gall-former species, inhabiting either of two closely related oak species, express strong preferences for their natal host plant species. Our results are consistent with partial habitat isolation evolving in these gall wasps as a by-product of adaptation to different hosts, as has now been demonstrated to be an important component to reproductive isolation in a number of plant–insect systems (*Timema* stick insects [50], leaf beetles [7], [54], pea aphids [55], ladybird beetles [56], *Rhagoletis* fruit flies [6], and *Eurosta* galling flies [49]).

An underlying assumption of our measurement of host preference is that the time spent on a host plant is correlated with the gall former’s subsequent mating and oviposition decisions, as has been shown in other host-associated and ecologically divergent insect populations [57]–[59]. We note that residence time as defined in our study is a conservative measure of host preference, as in two other specialist insect species that form host-associated populations, *Eurosta solidaginis* and *Rhagoletis pomonella*, insects are more likely to sit on the alternate host plant than to mate or oviposit on it [6], [57], [58]. Finally, for the six populations of *B. treatae* that we examined we have established the association between population level host preference and positive assortative mating [9], [10].

### Parallel Divergence and the Opportunity for Multiplicative Effects

In light of the regional overlap of the oak species [29], [60] and their associated gall wasp community (Table 1), our results suggest the hypothesis that habitat isolation may play a role in the evolution of reproductive isolation in this entire group [26]. Moreover, gall-former communities offer great systems to investigate parallel speciation in a tritrophic context [61]–[64], as global surveys find an average of approximately five parasitoid species per gall former (range: 1–120; [65]). Specifically here, the asexual generation of *B. treatae* is attacked by a community of up to 24 natural enemies [66], while the asexual generation *of D. quercusvirens* is attacked by a community of 10 natural enemies [38]. Thus, this study system also provides an opportunity to investigate the hypothesis of parallel sequential speciation for an entire community [62], [64].

### Conclusions

This study has documented parallel patterns of morphological and behavioral variation among host-associated populations of two gall-forming species. All populations of both gall-former species examined showed significant habitat preference, which can decrease the probability of mating between members of populations that reside on alternative host plants. These patterns support predictions of ecological speciation theory and are consistent with the hypothesis of host plant driven diversification in the species-rich and ecologically diverse Cynipidae.
